# The Novel Role of GDI2: A Mini-Review

**Published:** 2024-08-17

**Authors:** Y Wu, GY Chen

**Affiliations:** Department of Pediatrics, Children’s Foundation Research Institute at Le Bonheur Children’s Hospital, University of Tennessee Health Science Center, USA

## Abstract

GDP Dissociation Inhibitor 2 (GDI2) plays a crucial role in maintaining cellular homeostasis by regulating Rab GTPases involved in vesicular transport. This review highlights the importance of GDI2 in various biological processes, particularly embryonic development, apoptosis regulation, cancer, and immune responses. GDI2’s essential function in embryonic development is evidenced by the embryonic lethality observed in GDI2 knockout mice. In apoptosis, GDI2 is implicated in the caspase pathway, influencing cell survival and death. In cancer, dysregulation of GDI2 contributes to altered tumor cell-macrophage interactions, promoting inflammation and metastasis, with GDI2 acting as a metastasis suppressor in certain cancers. Furthermore, GDI2’s role in immune responses, particularly during bacterial infections, and its potential therapeutic implications in conditions like Alzheimer’s disease are explored. This review emphasizes the need for further research to elucidate the molecular mechanisms of GDI2 and its potential as a therapeutic target in developmental disorders, cancer, and immune-related diseases.

## Introduction

GDP Dissociation Inhibitor (GDI) binds to Rab GTPase in the GDP-bound inactive form to retrieve it from the cell membrane and maintain a soluble pool of inactive protein [1]. To date, more than 70 mammalian Rab proteins have been identified [2]. Some Rab proteins are expressed in specific tissues or stages of development, while others are ubiquitously expressed [2]. Each Rab protein has a characteristic subcellular distribution [3]. The GDI family includes GDI1 and GDI2 proteins. GDI1 is expressed primarily in neural and sensory tissues, whereas GDI2 is ubiquitously expressed [4]. The role of GDI2 protein in regulating Rab GTPases which are involved in vesicular transport within cells has been extensively studied [2,5–7]. The importance of GDI2 has been highlighted in various biological processes [2,6], including embryonic development and immune responses [8]. Recent studies have demonstrated that GDI2 is essential for normal embryonic development, with complete loss of GDI2 resulting in early embryonic lethality [8]. This review aims to discuss the novel findings from recent studies on GDI2, particularly its role in embryonic development, apoptosis regulation, and its implications in cancer and immune responses.

## GDI2 and Embryonic Development

GDI2’s essential role in embryonic development has been recently studied [8]. *Gdi2* is essential for embryonic development. One functional *Gdi2* allele is sufficient for murine embryonic development, but complete loss of *Gdi2* leads to embryonic lethality, with Gdi2^−/−^ embryos showing developmental retardation as early as E10.5 and no viable embryos detected after E14.5. Histological analyses revealed extensive cell death and apoptosis in Gdi2^−/−^ embryos, indicating that GDI2 is critical for maintaining cellular homeostasis during development [8]. In additional, GDI2 is a critical regulator of Rab GTPases influencing a range of cellular processes, including vesicular trafficking, cell signaling, and membrane dynamics [1,2,6,9,10], which may also be responsible for embryonic development. Continued research into GDI2’s function in embryonic development will enhance our understanding of developmental biology and potentially lead to new therapeutic approaches for related disorders.

## Apoptosis and GDI2

Apoptosis is a fundamental process for normal development of multicellular organism and is important in the regulation of the immune system, normal morphogenesis and maintenance of homeostasis. For example, non-functional or autoreactive lymphocytes are eliminated through apoptosis. Fas, a member of the Tumor Necrosis Factor Receptor (TNFR) family, can trigger cell death and is essential for lymphocyte homeostasis. GDI2 is a target for caspase cleavage upon apoptotic induction by Fas [11,12]. GDI2 knockdown inhibitors the cell cycle and promotes apoptosis in prostate cells [13]. GDI2 deficiency results in increased apoptosis in embryonic tissues. This was evidenced by extensive TUNEL-positive cells and cleaved caspase-3 staining in Gdi2^−/−^ liver sections [8]. Apoptosis is a fundamental process for normal development and homeostasis, and GDI2 appears to regulate apoptosis *via* the caspase pathway. The exact molecular mechanisms by which GDI2 influences apoptosis are still being explored, but it is suggested that GDI2 may interact with various signaling pathways that control cell death and survival.

## GDI2 in Cancer

In tumor cells, loss of GDI2 alters tumor cell-macrophage receptor crosstalk to enhance both local inflammation and tumor cell invasion and growth [14], leading to secretion of inflammatory cytokines by macrophages that promote metastatic growth of tumor cells. Dysregulation of GDI2 has been reported in many different cancers, including pancreatic carcinoma [15], ovarian cancer [16,17], gastric cancer [18], thyroid carcinoma [19], hematopoiesis and leukemogenesis [20] and esophageal squamous cell carcinoma [21]. GDI2 has also been identified as a suppressor of bladder cancer metastasis [22]. Reduced expression of GDI2 is associated with decreased survival of bladder cancer patients [23]; Restoring GDI2 expression has been shown to suppress metastasis without affecting primary tumor growth in animal models or growth in culture [14,22,24,25]. GDI2 appears to suppress metastasis by modulating GTPase signaling [26].

## GDI2 and Immune Response

Recent findings suggest that GDI2 plays a role in the immune response during bacterial infections [8]. Under normal conditions, GDI2 binds to the ITIM domain of Siglec-G, whereas Rab1a is recruited to the ITIM domain during bacterial infection. This interaction indicates that GDI2 and Rab1a may regulate immune responses through the ITIM domain [27]. However, the regulation of the inflammatory response by GDI2 *in vivo* remains to be fully elucidated. Studies involving Gdi2^+/−^ and wild-type mice challenged with Lipopolysaccharide (LPS) showed no significant differences in cytokine production and survival, suggesting that one functional GDI2 allele is sufficient for immune response during bacterial infection. Since loss of GDI2 alters tumor cell-macrophage receptor crosstalk to enhance both local inflammation and tumor cell invasion and growth [14], leading to secretion of inflammatory cytokines by macrophages. Future studies should focus on GDI2 deficient-macrophage by taking the conditional knockout strategies. Most recently, neuron-specific knockout of GDI2 alleviates neurodegeneration and memory loss in the 5xFAD mice model of Alzheimer’s disease [28], an autoimmune disease, in which the brain’s immune system mistakenly attacks brain cells [29,30].

## Molecular Mechanisms and Future Directions

The molecular mechanisms by which GDI2 regulates embryonic development, apoptosis, and immune responses are complex and involve multiple signaling pathways. Future research should focus on elucidating these mechanisms in detail. Conditional knockout strategies and advanced molecular techniques will be essential for uncovering the specific pathways and interactions mediated by GDI2.

## Conclusion

GDI2 is a critical regulator of Rab GTPases, playing essential roles in embryonic development, apoptosis regulation, and immune responses. Its dysregulation is associated with various cancers, highlighting its importance in maintaining cellular homeostasis and normal physiological functions. Further studies are needed to fully understand the molecular mechanisms underlying GDI2’s functions and its potential as a therapeutic target in cancer and immune-related diseases.
